# Combining implementation and data sciences to accelerate evidence integration into healthcare – ImpleMATE

**DOI:** 10.12688/f1000research.169999.2

**Published:** 2026-08-12

**Authors:** Jeffery Chan, Janna Hastings, Gabriella Tiernan, Jeannie Paterson, Nigel Lovell, Luc Betbeder-Matibet, Patrick Kin Man Tung, Sarah Pink, Debora Lanzeni, Thomasina Donovan, Andrew Milat, Louisa Jorm, Carolyn Mazariego, Chi Zhang, Guillaume Fontaine, Stephanie Best, Georgina Kennedy, Susan Michie, Frank Lin, The ImpleMATE Network, Natalie Taylor

**Affiliations:** 1 Implementation to Impact (i2i), School of Population Health, Faculty of Medicine and Health, University of New South Wales, Sydney, New South Wales, Australia; 2 Institute for Implementation, Science in Health Care, University of Zurich, Zurich, Switzerland; 3 School of Medicine, University of St. Gallen, St. Gallen, Switzerland; 4 Swiss Institute of Bioinformatics, Lausanne, Switzerland; 5 Centre for AI and Digital Ethics, The University of Melbourne, Melbourne, Victoria, Australia; 6 Graduate School of Biomedical Engineering, Faculty of Engineering, University of New South Wales, Sydney, New South Wales, Australia; 7 Tyree Institute of Health Engineering (IHealthE), University of New South Wales, Sydney, New South Wales, Australia; 8 Research Technology Services, University of New South Wales, Sydney, New South Wales, Australia; 9 Emerging Technologies Lab, Monash University, Melbourne, Victoria, Australia; 10 Department of Design, Monash University, Melbourne, Victoria, Australia; 11 Australian Centre for Health Services Innovation and Centre for Healthcare Transformation, School of Public Health and Social Work, Faculty of Health, Queensland University of Technology, Brisbane, Queensland, Australia; 12 Agency for Clinical Innovation, Sydney, New South Wales, Australia; 13 School of Public Health, University of Sydney, Sydney, New South Wales, Australia; 14 Centre for Big Data Research in Health, University of New South Wales, Sydney, New South Wales, Australia; 15 Ingram School of Nursing, McGill University, Montréal, Canada; 16 Centre for Clinical Epidemiology, Lady Davis Institute for Medical Research, Montréal, Canada; 17 Centre for Implementation Research, Ottawa Hospital Research Institute, Ottawa, Canada; 18 Australian Genomics, Melbourne, Victoria, Australia; 19 Melbourne School of Health Sciences, Faculty of Medicine, Dentistry and Health Sciences, The University of Melbourne, Melbourne, Victoria, Australia; 20 Faculty of Medicine and Health, University of New South Wales, Sydney, New South Wales, Australia; 21 Ingham Institute of Applied Medical Research, Liverpool, Sydney, New South Wales, Australia; 22 Maridulu Budyari Gumal (SPHERE) Cancer Clinical Academic Group, Sydney, New South Wales, Australia; 23 Centre for Behaviour Change, University College London, London, UK; 24 Department of Medical Oncology, Prince of Wales Hospital, Sydney, New South Wales, Australia; 25 Garvan Institute of Medical Research, Sydney, New South Wales, Australia; 26 NHMRC Clinical Trials Centre, University of Sydney, Sydney, New South Wales, Australia

**Keywords:** Artificial intelligence, data science, implementation science, natural language processing, large language model, healthcare

## Abstract

**Background:**

The translation of research evidence into routine healthcare practice is often slow and inconsistent, even timely implementation can significantly improve patient outcomes. While implementation science offers strategies to close this gap, current approaches are frequently manual, fragmented, and poorly integrated within healthcare systems. To address these challenges, we propose ImpleMATE – an AI-assisted implementation science platform designed to streamline implementation efforts. Grounded in the Learning Health System (LHS) model, ImpleMATE aims to establish a continuous, data-driven cycle of learning and improvement in implementation practice.

**Methods:**

ImpleMATE will be developed through a co-design and co-production approach rooted in human-centred design principles. Development will proceed through four key activities: (1) establishing a data processing pipeline and building an implementation-focused ontology; (2) creating and validating an AI system to extract implementation knowledge, structure the ontology, and support implementation solution delivery; (3) designing an interactive web application to deliver AI-assisted decision support and streamline implementation processes; and (4) developing an evaluation framework to assess platform’s effectiveness and plan for national integration. These activities align with three core components of the LHS model: converting data into knowledge, translating knowledge into practice, and feeding implementation process and outcome data back into the system for continuous learning. The platform will be underpinned by strong ethical and governance frameworks to ensure data privacy, transparency, and responsible AI use.

**Discussion:**

ImpleMATE aims to transform the adoption of evidence-based innovations in healthcare by embedding trustworthy AI into the core of implementation practice. Through the integration of structured ontologies, real-time AI reasoning, and an interactive user interface, the platform will provide tailored solutions to support implementation efforts. Designed as a dynamic learning system, ImpleMATE will evolve with user input and real-world data, offering a scalable, ethically grounded solution to accelerate and enhance implementation across healthcare settings.

## Background

Health research has progressed at an unprecedented pace. These advances have led to a deeper understanding of disease mechanisms, ^1^ the development of more targeted therapies, ^2^ and the generation of high-quality evidence for improving health outcomes. ^3^ Despite rapid scientific advances in health and medical research, the integration of evidence-based innovations into routine healthcare remains a persistent challenge. ^4, 5^ Evidence indicates that it can take on average between 7-17 years for evidence to be fully integrated into standard care. ^6– 8^ The slow uptake of research findings into practice is particularly problematic in fast-moving fields like cancer genetics and genomics where breakthroughs have transformed cancer care ^5, 9^ and enabled more precise diagnostics and targeted therapies that can improve survival and reduce mortality. ^10– 12^ This implementation gap significantly impedes timely and effective translation of emerging evidence into improved patient outcomes. The integration of breakthrough into everyday clinical practice continues to face complex, multifaceted barriers. ^4, 13– 21^

Implementation science has emerged as a key discipline to bridge this evidence-to-practice gap. Evidence-driven implementation strategies can drive effective system and practice change, improve health outcomes, and reduce costs. ^22– 24^ For example, tailored implementation strategies including actions plans, education, use of clinical opinion leaders, local, external, and country champions for site support, audit and feedback, and reminder strategies were used to implement nurse-initiated protocols to manage fever, hyperglycaemia, and swallowing after stroke in 64 hospitals across 17 countries, leading to significant reductions in death and disability. ^25^ The effectiveness of tailored implementation strategies, addressing context-specific implementation barriers and facilitators, is further supported by other large scale systematic reviews and meta-analyses. ^26, 27^ For example, a systematic review of 28 studies indicated social support was a key strategy associated with successful implementation of guidelines to ensure patients at high risk of hereditary cancer receive genetic testing and counselling. ^28^ Implementation science can inform us about contextual factors including barriers, facilitators, and strategies that influence the adoption, adaptation, and sustainability of evidence-based interventions in real-world settings.

In addition to identifying factors associated with successful implementation, optimising implementation effectiveness beyond a single study requires identifying the mechanisms of change: the processes through which implementation strategies impact care delivery. ^29^ For example, through mediation analysis within a randomised controlled trial to improve measurement-based care implementation in youth mental health, it was found that a leader-focused implementation strategy improved implementation climate by increasing leaders’ use of implementation leadership, and improved clinician fidelity to a clinical intervention by enhancing implementation climate. ^30^ In the genetics space, a mixed-methods process evaluation was conducted on seven Australian hospitals, alongside a hybrid type III trial to improve Lynch syndrome detection. The study found that tailored theory-driven strategies, including education, training, and MDT strategies, may better support Lynch syndrome detection practices. ^31^

Whilst these complex trials of strategies and analyses of mechanistic effects help to advance the science of implementation, this work relies heavily on collection and manual data analysis of diverse data sources such as implementation literature, live project data, surveys, interviews, meeting records/documentation, and outcome data. Synthesising this wealth of information across thousands of studies to understand patterns of implementation success for different interventions and contexts is an infeasible challenge. Currently, identifying and applying effective implementation strategies is often a manual and resource-intensive process. These approaches are not only inefficient and hindered by the underutilisation of existing knowledge, but also susceptible to bias and inconsistency, limiting their scalability and impact. As a result, health systems often duplicate efforts, reinvent strategies, and fail to capitalise on prior learnings. ^32– 35^

Implementation science also lacks dedicated infrastructure within health systems to systematically support the adoption of evidence-based practices. ^36, 37^ Missing infrastructure includes effective and safe data sharing capabilities, local, national, and international communities of practice, availability of practical educational resources, and access to implementation expertise for training and coaching support. ^38– 40^ There is an urgent need for a more scientific, structured, and integrated approach to make health system implementation smarter. If we do not act, we risk continuing to waste time rehashing unsuccessful implementation approaches, with healthcare delivery continuing to lag behind the pace of innovation, leaving patients without timely access to the full benefits of cutting-edge research.

To address these persistent challenges, we propose an implementation research and practice platform: ImpleMATE – combining *ImpleMentation and dATa sciences to advance the speed of evidence integration into hEalthcare.* ImpleMATE harnesses the power of artificial intelligence (AI) and integrates expert-guided feedback mechanisms to directly address these interrelated implementation research and practice, and health system inefficiencies through automatic knowledge (i.e., concept) curation, synthesis, transfer, and continuous improvement.

## The potential of combining implementation and data sciences

Implementation science provides the theories, frameworks, and tools to support practical evidence translation and help advance our understanding how and why interventions succeed or fail in real-world settings, ^41^ while data science offers the computational power and analytical techniques to process large, complex datasets efficiently. Combining these two disciplines presents an opportunity to accelerate the implementation process, reduce inefficiencies, and minimise human error in decision-making processes.

A data science approach can support the design and development of an end-to-end data pipeline that automates the data collection from diverse sources, performs data screening, cleansing, preprocessing, storage, analysis, and retrieval. ^42^ This pipeline can accommodate both structured (e.g., survey data, practice change data) and unstructured data (e.g., literature, interview data). Data screening processes validate the extent to which the incoming data falls within the project scope. Keyword matching, topic modelling, and AI-based semantic analysis can be used for this purpose. ^43, 44^ Data cleansing involves handling missing data, deduplication, and format standardisation, ensuring data quality and consistency. Preprocessing can include de-identification to comply with ethical and privacy standards, as well as indexing, which involves tagging or annotating key concepts, ^45^ such as clinical terms, interventions, and strategies, within the data. These concepts are organised using an ontology, which is a structured representation of knowledge that defines the concepts and their relationships. ^46^ Ontologies have been widely used in different fields, including behavioural science, ^47– 49^ enabling consistent interpretation of data, and supporting more accurate and meaningful data integration, retrieval, and analysis.

Depending on data type and volume, storage solutions may include relational databases, data warehouses, or cloud-based data lakes. Analytical models, including statistical analysis and machine learning algorithms, can be applied to uncover patterns, generate insights, and support context-specific decision-making. Predictive models can forecast the likely success of different implementation strategies by drawing on historical data and contextual variables. ^50^ Information retrieval techniques can help ensure that users access relevant findings through dashboards or query interfaces, delivering actionable insights when and where they are needed. This systematic approach enhances data usability, reproducibility, and scalability in implementation research and practice.

The integration of AI, particularly natural language processing (NLP), within this data pipeline offers additional potential. NLP enables automated identification and extraction of implementation-relevant concepts and their interrelationships from unstructured data sources such as literature, reports, and meeting notes. ^48, 51– 53^ Transformer-based large language models (LLMs), such as encoder-only models like Bidirectional Encoder Representations from Transformers (BERT), ^54^ or decoder-only models such as Generative Pre-trained Transformer (GPT), ^55^ Mistral, ^56^ and LLaMA, ^57^ can perform this task with potentially high accuracy. ^51^ Depending on the availability of labelled data, these models can be fine-tuned to improve task-specific performance. Prompting strategies, such as zero-shot, ^58^ few-shot, ^59^ and chain-of-thought (CoT) prompting, ^60^ can be used to control and guide the models’ outputs.

NLP can also be leveraged to rapidly synthesise findings from large volumes of implementation literature. ^61^ By extracting and clustering key concepts, this can help to identify common implementation barriers, facilitators, and effective strategies across studies and contexts. The ability of NLP to understand semantic relationships between concepts, recognise synonyms, and interpret context allows for a more nuanced synthesis of evidence. For instance, NLP can cluster similar implementation determinants under shared categories, or link strategies to specific outcomes across different settings. ^62, 63^ However, the implementation landscape is inherently complex with interrelated, dynamic, and context-specific factors. Across the hundreds of thousands of reported implementation studies conducted in the past, present, and ongoing, clinical and research teams are striving to capture the wealth of information generated in real time. This information, collected on a spectrum from real-world quality improvement settings to rigorously designed implementation trials, is highly varied in terms of structure and quality, and often difficult to consolidate into a single, coherent data capture framework. To meaningfully address this complexity and generate reliable, actionable strategies, a platform that enables systematic and structured data capture from live implementation projects, integrated with NLP approach, holds significant promise. This kind of platform has the potential to enhance efficiency, enable real-time feedback and adaptation, and improve the accuracy and relevance of implementation insights. While AI is not a standalone solution and requires thorough testing and a robust governance framework for its own effectiveness and to mitigate harmful bias to optimise an implementation effort in comparison to standard approaches, the potential lies in helping to manage complexity, support timely decision-making, and ultimately improve the design and execution of evidence-informed implementation strategies.

As implementation science continues to evolve, integrating data science – including AI – offers a transformative approach to accelerating knowledge translation, avoiding redundant efforts, and systematically capturing and reusing implementation knowledge. This synergy has the potential to reshape how evidence is collected, synthesised and applied across healthcare systems.

## Aim and research questions

This project aims to pioneer the world’s first expert-guided and AI-assisted implementation science platform – ImpleMATE – to revolutionise implementation research and practice. ImpleMATE integrates AI with a continuous feedback and improvement mechanism inspired by the Learning Health System (LHS) model, ^64^ directly targeting key inefficiencies in implementation science and healthcare delivery. We will initially focus on cancer genetics and genomics, and once proof of concept is established, we will look to extend to other areas.

**Figure 1.**
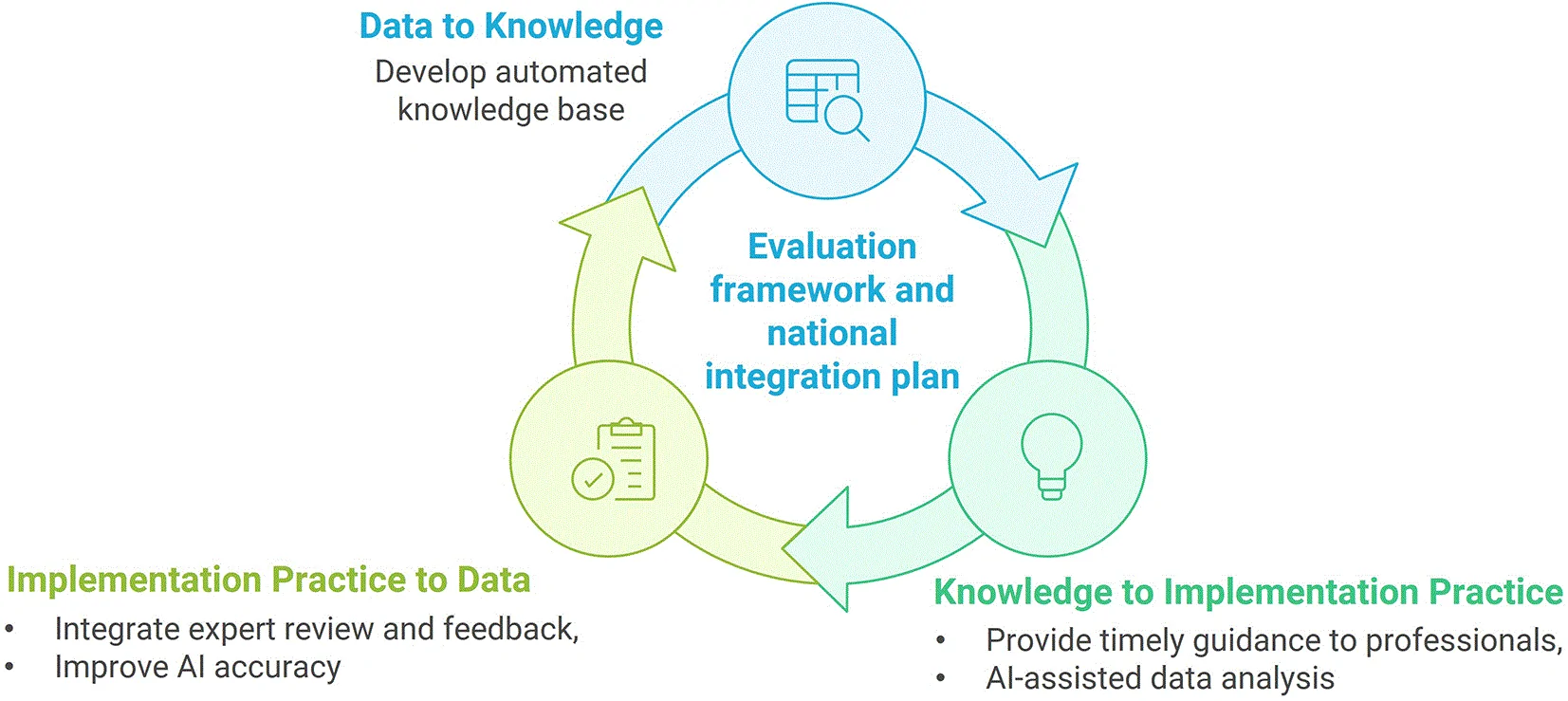
ImpleMATE learning and improvement cycle comprising the Data to Knowledge (D2K), Knowledge to Implementation Practice (K2IP), and Implementation Practice to Data (IP2D) components.

The LHS concept for ImpleMATE consists of three components – “Data to Knowledge”, “Knowledge to Implementation Practice”, and “Implementation Practice to Data” forming a learning and improvement cycle as shown in Figure 1. The research questions are posed around these three components, with evaluation and ongoing planning for health system integration at the core:

**Data to Knowledge (D2K) – knowledge curation, identification, extraction, and representation:**

1) What are the knowledge sources and the types of the sources?
2) How can implementation knowledge be curated from the sources?
3) What is the optimal approach for managing process data from multiple sources with varying formats?
4) What essential implementation-related information, entity interrelationships, data organisation, and representation are required in an ontology?
5) What governance frameworks are required to ensure testing, transparency, accountability, and ethical use of curated knowledge to ensure trust in the platform?
6) How do we identify and extract the implementation-related information using AI? In particular, which AI models should be used?
7) What is the accuracy of the identification and extraction tasks, and how can bias in these tasks be detected and mitigated?
8) What is the relevance of the curated knowledge, and how is this reliably measured?

**Knowledge to Implementation Practice (K2IP) – knowledge transfer and implementation process organisation through an interactive web application**:

1) Who are the target users and what is the best approach for effectively transferring knowledge to users through an interactive web application?
2) What components are included in an implementation process and how are the components to be organised in an interactive web application to drive optimal approaches to implementation?
3) How to effectively retrieve the most relevant information from an ontology based on a user’s query?
4) How should the retrieved information be synthesised using AI to answer the query?
5) How can an interactive web application be integrated with backend AI for real-time inference to support a live implementation effort?

**Implementation Practice to Data (IP2D) – feedback and improvement mechanism:**

1) Where in the platform should feedback loops be integrated and how can they improve implementation?
2) What should be included in the feedback loop?
3) How often should the feedback be provided?
4) How is the feedback process integrated into governance framework?

**System Evaluation for Integration (SE)**:

1) How can this platform be integrated into the existing healthcare system?
2) What should an evaluation framework for national rollout look like?

## Methods

Our approach to develop ImpleMATE combines (1) co-design: focusing on collaborative, human-centred design of solutions to address the pre-identified problem of the interrelated implementation science and health systems inefficiencies; and (2) co-production: focusing on operationalisation of the solutions informed by user experience. ^65– 68^ This process will actively engage stakeholders including clinicians, implementation scientists, change managers, healthcare professionals, consumers, decision-makers, and data scientists. To ensure robust project governance, three dedicated groups will be established: a Steering Committee, a Principle Setting End User Committee, and a System Integration Advisory Group.

**Figure 2.**
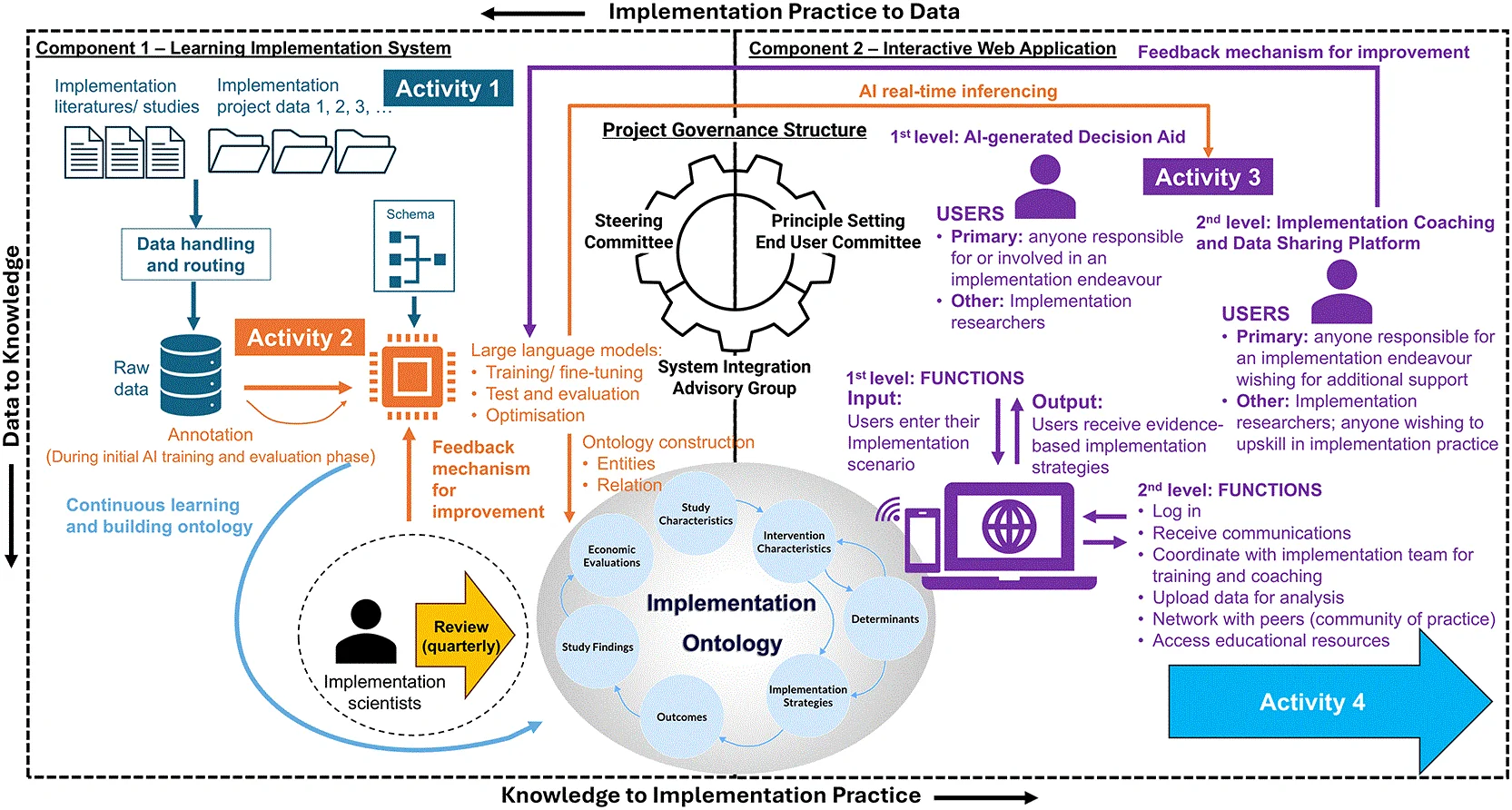
An overview of ImpleMATE structure – the four interconnected activities.

Development will proceed through four core activities aligned with the research questions: Activity 1 will address data handling and ontology design; Activity 2 will focus on AI model development; Activity 3 will involve web application creation; and Activity 4 will evaluate outcomes and develop a roadmap for national system integration. Activities 1 and 2 will form a core Component 1 – Learning Implementation System to enable continue learning of implementation knowledge, and Activity 3 will form a core Component 2 – Interactive Web Application (including both 1 ^st^ and 2 ^nd^ levels of support) to facilitate knowledge transfer. An overview of the ImpleMATE structure and its four activities is presented in Figure 2.

### Activity 1. Design data handling process to organise incoming data and a structured implementation ontology

Address research questions: D2K Q1-5

***Handle incoming data and ensure data compatibility with large language models (LLMs)***

We will focus on curating two types of data sources. The first data source will be published implementation literature: A data pipeline will curate implementation literature from PubMed research database through an application programming interface (API). The initial focus will be on published cancer genetics/genomics studies that used two key frameworks (collectively cited >9000 times for use in implementation studies, ^69– 71^ and recently combined ^72^) to support evaluation and fine-tuning of LLMs: the Theoretical Domains Framework (TDF) ^70^ for individual behaviour change, and the Consolidated Framework for Implementation Research (CFIR) ^73^ for organisational and policy factors affecting implementation. Studies using alternative frameworks, other healthcare areas, and/or with unstructured implementation data will be our next focus. To ensure the literature is within the scope, we will use two approaches: (1) keyword searches in titles and abstracts, and (2) topic modelling with both statistical and machine learning (ML) approaches such as Latent Dirichlet Allocation (LDA) ^74^ and BERTopic ^75^ with embeddings to capture semantic representation of the literature for topic classification and filtering. Regular meetings with clinicians and implementation scientists will guide keyword and topic selection. The second data source will be existing and ongoing project data (e.g., surveys, interviews, meeting records). Initially, this platform will maximise the use of available data and resources across existing cancer genetics/genomics implementation projects in our project team. The data will/has been stored in a secure institutional university data repository.

The collected data will come in various formats, including PDFs, text files, audio recordings, and spreadsheets. To standardise the input for AI processing and ensure compatibility across downstream tasks, all data will be converted into plain text. Prior to any further processing, we will apply and compare de-identification techniques to remove personally identifiable information, in accordance with the State and Federal Australian Privacy Laws. For literature data, segmentation techniques (e.g., using regular expressions) will be employed to identify and separate sections, ensuring the input stays within the token limits (i.e., amount of content LLMs can process) required by LLMs and adheres to their specific formatting requirements. For project data, we will assess and address missing data and duplication. This preprocessing step is critical to ensure data sanitisation, privacy compliance, and security, while also preserving the integrity and accuracy of the data representation when deploying LLM-based solutions.

The platform will initially operate within secure institutional computing infrastructure using locally hosted AI models to ensure that sensitive implementation project and health data remain within controlled organisational environments during development and evaluation. Future deployment to cloud-based environments will only occur where the infrastructure satisfies institutional security requirements, applicable privacy legislation, and established data governance frameworks.

***An implementation schema to guide the development of ontology***

To support structured knowledge extraction and synthesis, we will define a comprehensive implementation schema in collaboration with stakeholders. This schema will establish the scope of the ImpleMATE ontology by defining the key implementation science entities, their attributes, and their interrelationships required to represent implementation knowledge across healthcare settings. The ontology will initially focus on implementation concepts relevant to cancer genetics and genomics, while being designed as a modular and extensible framework that can progressively incorporate additional healthcare domains as new implementation knowledge becomes available through the Learning Health System cycle.

The schema will capture key entities, their interrelationships, and the overall data structure. It will include categories such as implementation study characteristics, intervention characteristics, implementation determinants (i.e., factors influencing implementation success), strategies, and outcomes, including costs. For instance, study characteristics may include the study setting, design, target population, and geographic location. Interventions may involve, for example, whole body MRI scanning to more accurately detect cancers in patients with LiFraumini syndrome. ^76^ Implementation determinants may include system level factors such as workflow integration, changes to existing approaches for cancer prevention in these patients, and access challenges for patients in remote locations, and individual clinician factors including remembering to use this new approach, skills to interpret results, and trust in the technology. These factors can be aligned with established implementation frameworks (e.g., TDF and CFIR), allowing for a structured identification of barriers and facilitators.

Implementation strategies may include specific methods (e.g., system mapping and workflow redesign, prompts, audit and feedback) used to promote the adoption and integration of interventions into practice. Outcomes (which can be aligned to frameworks such as Proctor’s Outcomes Framework ^77^) may include measures such as fidelity, acceptability, and sustainability, as well as service level, clinical and economic outcomes, including associated implementation costs. ^78, 79^ The schema will be developed through an iterative and collaborative process that involves gathering stakeholder feedback, refining entity definitions, and structuring the schema to reflect real-world implementation scenarios. This is to provide a structured framework for describing and managing an ontology to represent the implementation knowledge.

Throughout this process, stakeholders will be actively involved in reviewing and validating the framework, including assessments of inter-rater reliability to ensure consistency and clarity in the defined categories. Consumers will be engaged early to ensure that the ontology reflects patient priorities and is grounded in real-world healthcare experiences. This co-design approach ensures that the ontology is not only technically rigorous and accurate, but also practical, contextually meaningful, and sustainable over time.

The schema will be developed in alignment with the Open Biomedical Ontologies (OBO) Foundry principles, ^80^ ensuring the framework is interoperable, transparent, extensible, and adheres to accepted standards for ontology development. Where appropriate, implementation concepts will align with or reference established biomedical terminologies and ontologies and relevant OBO ontologies. This approach will promote consistency in terminology, facilitate interoperability with existing health information systems, and avoid duplication of established biomedical knowledge. It will also enable implementation knowledge generated through ImpleMATE to be integrated with existing healthcare data and evidence resources while maintaining compatibility with established knowledge standards.

The ontology will be refined iteratively throughout the project using both technical and expert-informed evaluation. Technical evaluation will include assessment of logical consistency, completeness, interoperability, scalability, extensibility, and structural integrity to ensure the ontology effectively supports AI-assisted knowledge extraction and retrieval across diverse implementation contexts. Expert evaluation involving implementation scientists, clinicians, healthcare managers, and end users will assess the accuracy, relevance, usability, and practical value of the ontology for supporting implementation research and implementation decision-making. Feedback from these evaluations will be incorporated into successive ontology versions as additional implementation knowledge is curated through the Learning Health System cycle.

**Figure 3.**
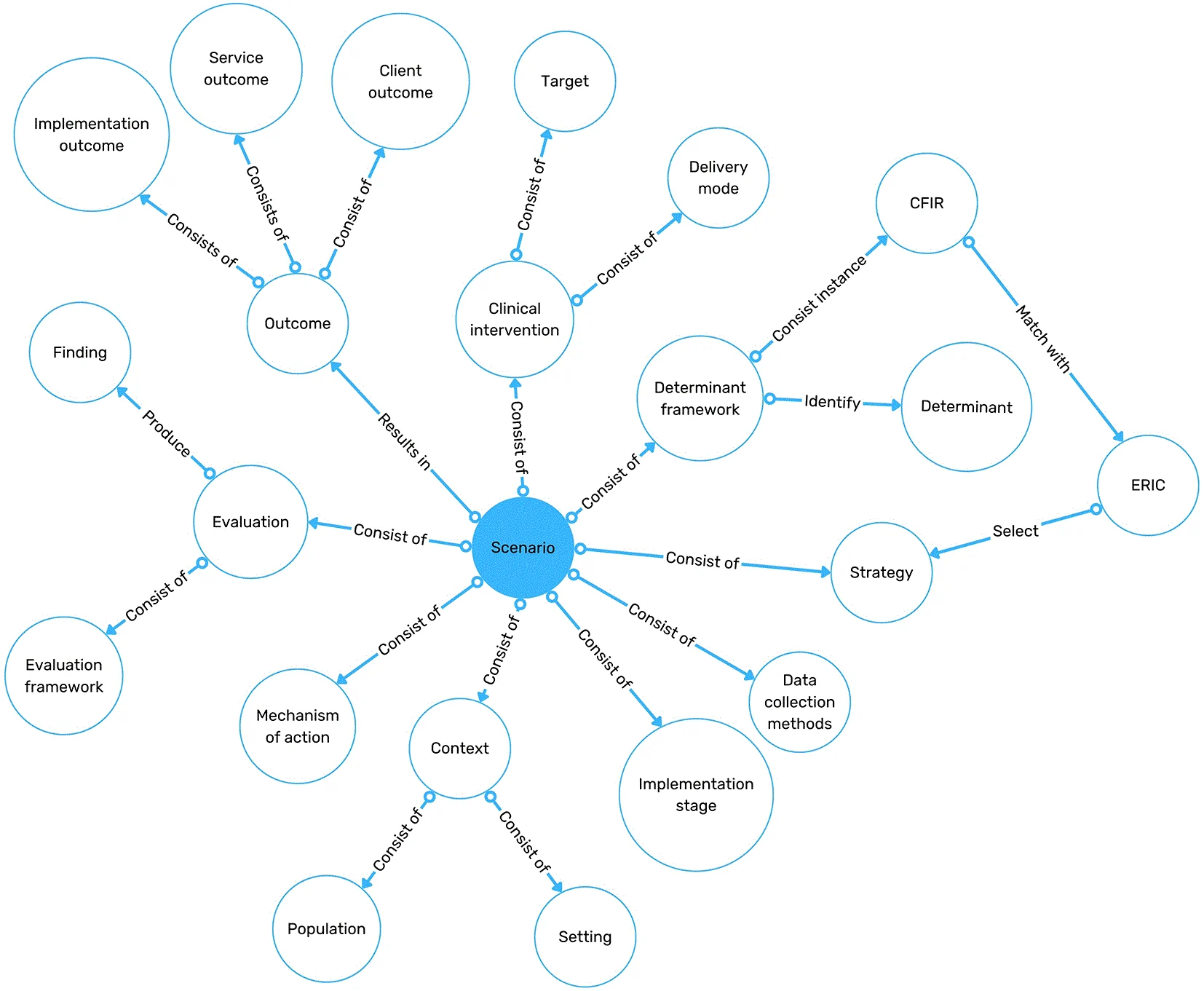
An example of implementation science ontology representing key entities, their attributes, and interrelationships.

The implementation schema will support the identification and organisation of information that addresses critical implementation questions about what works, why, in which settings, for whom, and at what cost. It will provide the knowledge foundation for AI-assisted evidence retrieval, implementation planning, and implementation decision support within ImpleMATE. An example of the implementation science ontology is illustrated in Figure 3.

***Ethical sharing of project data and responsible use of AI***

To develop a system that relies on both the sharing of research and live project data, and the synthesis of that data using AI, it is essential to establish a solid and sustained foundation for data privacy, security, and the responsible use of AI. To achieve this, we will establish a Principle Setting End User Committee, which will both inform the AI governance framework and act as the conduit to the wider group of relevant consumers, researchers, and clinicians. The End User Committee will coordinate and lead a series of workshop-style focus groups to present proposed data journey pathways and collaboratively explore approaches to optimise data access while maintaining privacy standards. The goal is to meet both legal requirements and public expectations, establishing and maintaining a social license for data use, and promoting an approach to AI that is fair, inclusive and trustworthy.

The focus groups will also explore and refine principles for AI transparency and accountability, ensuring that the AI technologies employed are understandable, explainable, and ethically guided. The End User Committee will remain engaged throughout the project to support the iterative design, development, and deployment of the platform, ensuring its ethical integrity is maintained during the development.

In addition, we will conduct targeted interviews to gather diverse stakeholder perspectives on how best to ethically manage and share implementation project data, and how to ensure the responsible application of AI in this domain. Insights from these qualitative investigations will be analysed to develop a set of ImpleMATE Ethics Principles. These principles will be reviewed with the committee to establish consensus and will inform the development of comprehensive data and AI governance frameworks. This ensures transparency in data processing, clarifies the full data journey, and supports accountability for how AI and analytics are used in the project, ultimately reinforcing public trust and supporting ethical innovation in implementation science.

To operationalise these governance principles, ImpleMATE will implement a secure data management framework that protects sensitive implementation project and health data throughout their lifecycle. All project data will undergo de-identification prior to AI processing, with direct identifiers removed or replaced in accordance with approved ethics protocols and institutional data governance requirements. Data will be encrypted during transmission using secure communication protocols and encrypted at rest within secure institutional infrastructure. Access to project data, AI models, and system administration functions will be restricted through role-based access controls and user authentication, with audit logs maintained to record system access and data use to ensureing that users can only access information appropriate to their responsibilities. Data retention, archival, and disposal will follow institutional policies, ethics approvals, and the State and Federal Australian Privacy Laws.

Given the sensitivity of implementation project data, particularly where genomic or other health information may be involved, ImpleMATE will prioritise locally hosted AI models operating within secure computing environments to minimise unnecessary transfer of sensitive information to external services. Where future collaborations require data sharing across organisations, only appropriately governed, de-identified datasets will be shared under approved data sharing agreements, with all data access governed by institutional ethics approvals and established governance arrangements.

### Activity 2. Develop and validate an AI system to automate ontology creation and deliver real-time solutions to implementation challenges

Address research questions: D2K Q6-8, IP2D Q1-4

***AI model selection and implementation concept extraction***

To enable automated, real-time identification and mapping of implementation science concepts, we will develop and validate an AI-assisted system that aligns with the ontology defined in Activity 1. Implementation science experts will first analyse preprocessed data to create a gold-standard dataset by manually annotating concepts including study characteristics, barriers, facilitators, strategies, and outcomes defined in the ontology framework. Documents will be purposively selected to represent a broad range of implementation studies, healthcare settings, intervention types, and implementation contexts to ensure diversity in implementation concepts and contexts. Eligible documents will include peer-reviewed implementation studies and systematic reviews, together with implementation project data initially focused on cancer genetics and genomics. Documents that lack sufficient implementation content or contain incomplete information will be excluded.

Annotation will be undertaken using a standardised annotation guideline developed from the implementation ontology. The guideline will define implementation concepts, annotation rules, inclusion and exclusion criteria, and representative examples to promote consistency across annotators. Prior to full annotation, a pilot annotation exercise will be conducted to refine the guideline and ensure consistent interpretation of the annotation criteria.

Each document will be independently annotated by at least two implementation science experts. Inter-rater agreement will be assessed using Cohen’s kappa coefficient for categorical annotations to evaluate annotation consistency before finalising the gold-standard dataset. Any disagreements will be resolved through discussion and consensus, with adjudication by a senior implementation scientist where consensus cannot be reached. The final consensus annotations will constitute the gold-standard dataset for AI model development and evaluation.

An initial gold-standard dataset comprising 1,000 documents will be used to evaluate transformer-based models for their capacity to extract and structure relevant information. Given the diverse and heterogeneous nature of implementation science concepts, ranging from study characteristics and outcomes to more nuanced elements such as barriers, facilitators, and strategies, this dataset will support the exploratory phase of initial model validation and performance comparisons across concept categories.

Our approach will involve two core development pathways. First, we will test and fine-tune a concept extractor such as BERT model for Named Entity Recognition (NER) and Relation Extraction (RE), enabling the system to accurately identify discrete implementation concepts and their relationships. Second, we will collaboratively design and evaluate prompt engineering techniques tailored to pretrained, locally hosted transformer models such as Mistral ^56^ and LLaMA. ^57^ These models will be explored using a range of strategies, including zero-shot and few-shot learning ^58, 59^ as well as CoT reasoning, ^60^ to determine their effectiveness in extracting relevant information from varied, context-rich data sources. Examples of prompts for extracting implementation concepts are shown in Table 1. The use of locally hosted models within secure institutional computing infrastructure ensures that sensitive health and implementation project data remain within controlled organisational environments, reducing unnecessary transfer of information to external services while supporting compliance with institutional governance requirements, privacy legislation, and ethical standards. ^81^

**Table 1.** Prompt examples for building the ontology and assisting implementation concept identification and extraction.

Implementation concepts	Prompts
Settings	You are an expert in health system analysis. Read the case report and identify the settings in which the intervention or strategy was implemented. The settings could include the type of institution (e.g., hospital, community health center), the geographic location (e.g., city, rural area), the organisational context (e.g., public or private sector), and any other relevant environmental factors that describe where the implementation occurred. Please list and explain each identified setting.
Implementation barriers	You are an implementation scientist. Read the case report and identify any barriers to the successful implementation of the intervention or strategy discussed. These barriers can be related to organisational, systemic, financial, technological, or human factors, among others. Please list and explain each identified barrier. Return NA if no barrier is identified.
Implementation strategies	You are a clinical nurse consultant. Read the case report and identify the strategies that were used to overcome challenges or improve the implementation of the intervention or strategy. These strategies can include actions related to enhancing clinical workflows, engaging multidisciplinary teams, optimising resource use, and more. Please list and explain each identified strategy.

To promote trustworthy AI, ImpleMATE will adopt an evidence-grounded approach in which implementation concept extraction and AI-generated outputs are informed by the curated implementation ontology and supporting implementation evidence rather than relying solely on the reasoning capability of the underlying LLM. Where feasible, extracted implementation concepts, summaries, and recommendations will be linked to the corresponding ontology concepts and supporting evidence used during inference, enabling users to understand the rationale underpinning AI-assisted outputs. This approach is intended to improve transparency, reduce the likelihood of hallucinations, and ensure that AI-generated outputs remain aligned with established implementation science knowledge.

As LLM models learn from increasing volumes of annotated data, their performance in identifying and extracting implementation concepts is expected to improve, enabling them to support the progressive development and refinement of the ontology. Importantly, ontology updates and knowledge curation will remain subject to expert review through a human-in-the-loop process rather than being incorporated automatically. This continuous learning process will contribute to the dynamic evolution of the ontology and enable the system to deliver real-time, context-specific implementation insights. These insights will form the backbone of the interactive web application developed in Activity 3, allowing users to query and retrieve relevant strategies and evidence-based recommendations efficiently, and supporting the implementation process.

To ensure accuracy and practical relevance, the outputs of each model will be systematically compared against expert-annotated datasets. Through this evaluation process, we will determine whether a single model or an ensemble approach provides the most reliable and generalisable performance. Implementation scientists will review AI-generated outputs throughout model development to identify incorrect, incomplete, or potentially biased implementation concepts and recommendations. Feedback from these reviews will inform refinement of prompts, ontology content, and evidence curation to improve AI performance across diverse implementation contexts. This effort will also serve to validate the overall feasibility and robustness of a computational curation pipeline, demonstrating its potential to support scalable, automated knowledge synthesis in implementation science.

***AI performance evaluation metrics, quality assessment and monitoring***

To ensure the reliability, accuracy, and continuous improvement of the AI system, we will implement a structured framework for evaluation and quality monitoring. The annotated dataset will be randomly divided into training (70%), validation (15%), and independent test (15%) subsets while maintaining representation across implementation settings. A reserved validation dataset from the gold-standard dataset will be used for prompt optimisation, while the independent test dataset will serve as the ground truth for final model evaluation. Model performance will be compared against expert manual annotation. Standard performance metrics, including precision (the proportion of correctly identified entities among those predicted), recall (the proportion of relevant entities successfully identified), and F1-score (the harmonic mean of precision and recall), will be used to quantitatively assess the accuracy and effectiveness of concept extraction. Performance evaluation will also include semantic similarity, agreement with expert annotations, and, where appropriate, confidence intervals generated through bootstrapping to quantify the robustness and uncertainty of performance estimates.

Beyond this surface-level matching, we will assess semantic equivalence between AI-generated outputs and expert annotations to account for cases where the same concept is expressed using different wordings. This semantic comparison ensures that meaning is preserved even when the linguistic form differs, which is especially important for interpreting complex implementation science data. The AI system’s adaptability to new or previously unseen concepts will be closely monitored. Performance monitoring will also assess the consistency of concept extraction across diverse healthcare settings, intervention types, implementation contexts, and populations to identify potential systematic bias. Where variations in performance are identified, implementation scientists will investigate the underlying causes and refine prompts, ontology content, or evidence curation processes as appropriate to improve robustness and generalisability.

During model development, we will systematically test multiple prompt formulations, comparing their outputs against the validation dataset using the defined metrics. In addition to performance, prompt quality will be assessed based on relevance and the occurrence of hallucination, which is defined as content not grounded in the data source. An iterative refinement strategy will be employed, using the LLMs to suggest and optimise its own prompts to generate candidate prompts. The final prompt selection will be based on both effectiveness and generalisability across diverse implementation science contexts. Where feasible, AI-generated outputs will be accompanied by references to the implementation evidence and ontology concepts that informed the response. This evidence-grounded approach will improve transparency, enabling users to understand the basis of AI-assisted implementation recommendations while supporting expert validation of generated outputs.

Regular qualitative assessments will be conducted to identify emerging themes, assess the specificity and generalisability of the model, and highlight areas for improvement. Manual reviews of the evolving ontology, led by implementation scientists, will ensure that AI-generated outputs remain consistent with domain expertise and responsive to real-world practice needs. Human-in-the-loop review will be incorporated throughout ontology development, implementation concept extraction, knowledge curation, and platform evaluation to ensure that AI-generated outputs are validated before incorporation into the implementation knowledge base. These evaluations will inform targeted adjustments, such as fine-tuning or prompt control, through a feedback mechanism integrated into the ImpleMATE structure and embedded within the broader governance framework, ensuring that insights from performance monitoring and expert review are systematically incorporated into decision-making processes. Pragmatic success criteria will include achieving expert-level agreement for key implementation concepts and demonstrating substantial reductions in manual coding time without compromising coding quality.

Importantly, ImpleMATE is intended to support implementation decision-making rather than provide clinical diagnosis or direct patient management recommendations. Final implementation decisions will remain the responsibility of implementation teams and healthcare professionals. This iterative approach will drive continuous system improvement, ensuring the AI remains robust, accurate, and contextually relevant in addressing real-world implementation challenges.

### Activity 3. Create and test an interactive web application to support implementation decision-making and streamline implementation processes

Address research questions: K2IP Q1-5, IP2D Q1-4

***An AI-assisted decision aid, and an implementation coaching and data sharing platform***

The interactive web application developed in this project will provide two integrated levels of support for implementation practice. The target users include implementation researchers, clinicians, change managers, and other professionals engaged in evidence translation efforts across healthcare settings. The 1 ^st^ level will offer an AI-assisted decision aid, while the 2 ^nd^ level will deliver a more advanced and comprehensive implementation coaching and data sharing platform.

An AI-assisted decision aid – This will allow users to input queries related to their implementation context or challenges they are facing. For example, users might ask, “How can I implement X intervention in my setting?” or “Which strategies could help me address a specific barrier?” The system will analyse each query through a text processing pipeline, matched against the ontology created in earlier stages of the project. The ontology will serve as a knowledge base, enabling the retrieval of relevant concepts and their contextual relationships. The semantic meaning of the query will be interpreted by the local LLM, which synthesises the retrieved information and generates a tailored, evidence-based response that aligns with the user’s context. Basic and comprehensive queries will be prepared and regularly submitted to the decision aid for testing to ensure relevance of the retrieved concepts and relationships, as well as accuracy of the AI-generated responses. This supports iterative improvement in the model’s performance and the quality of outputs.

An implementation coaching and data sharing platform – this provides more comprehensive support that functions as a central hub for implementation professionals and clients. It will enable users to securely log in, communicate with implementation support teams, access coaching and training resources, and share data collected during real-world implementation projects. Users will be able to upload a wide range of data, including surveys, meeting records/documentation, process maps, focus group transcripts, cost analyses, and health system outcome metrics. The platform will support implementation scientists to analyse this data through AI-assisted tools such as automatic qualitative coding and strategy mapping, matched against established implementation frameworks like CFIR and ERIC. This functionality will offer deeper insights into the determinants, strategies, and outcomes relevant to their specific projects.

The platform will include a feedback mechanism that allows users to report perceived inaccuracies in AI-generated responses. These observations will inform further refinement of the system through prompt engineering or fine-tuning of the underlying models. The data and insights generated through this platform will be fed back into the Learning Implementation System, expanding and continuously improving the ontology. The web application itself will be initially developed and validated in a secure local environment before being deployed to a secure web server. The LLM will be accessed through a secure and encrypted channel to ensure data privacy and compliance with relevant legal and ethical standards.

**Figure 4.**
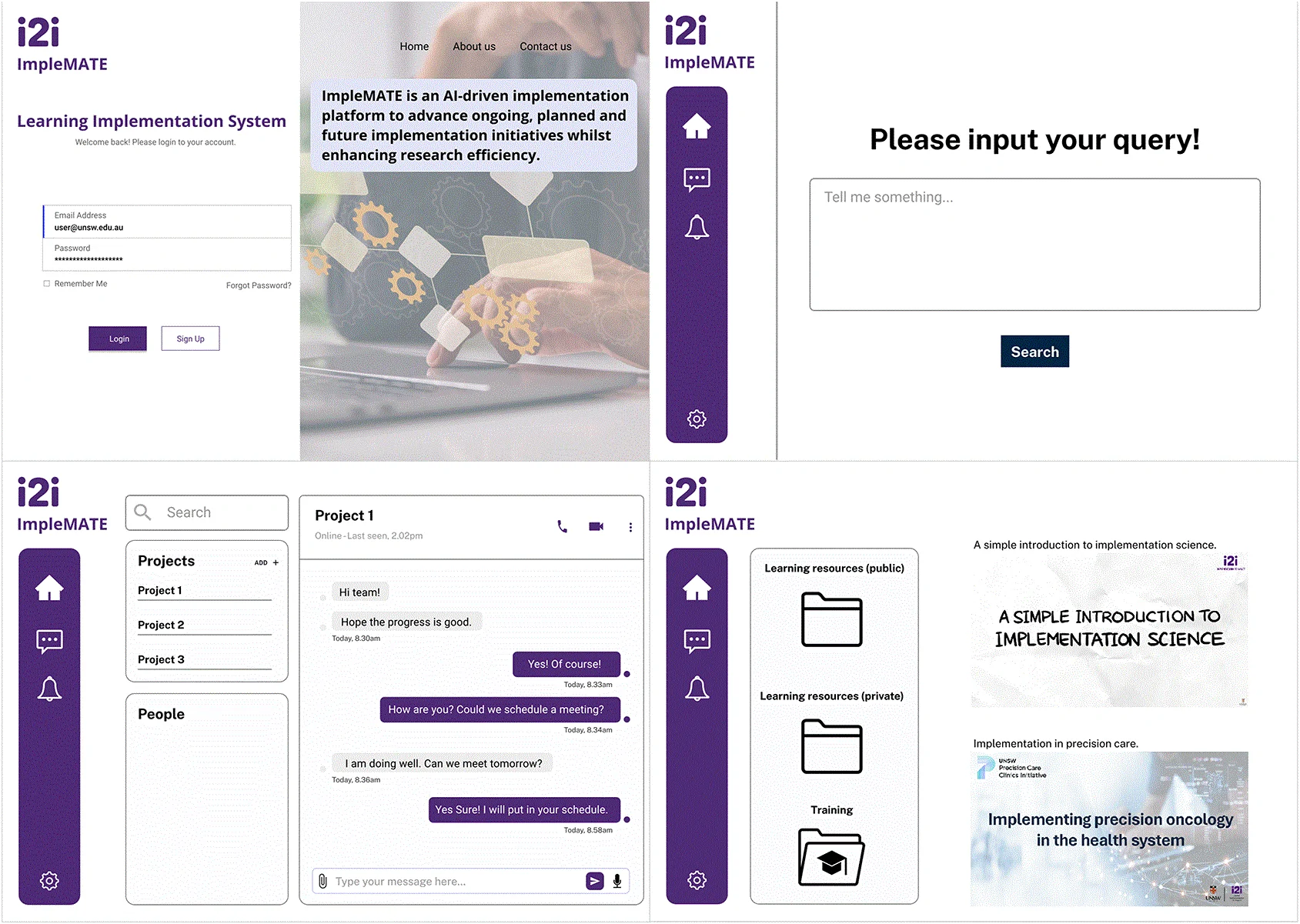
Prototype ImpleMATE web application supporting AI-assisted implementation decision support, coaching, and data sharing.

To support broader scalability, the platform will be designed with flexible access levels to accommodate the varying needs of different user groups. This architecture ensures that the system can serve as both a lightweight decision aid for individual users and a full-featured collaborative platform for research teams and institutional partners. Examples of user scenarios and the levels of support available to them are described in Table 2. Examples of the web application is shown in Figure 4.

**Table 2.** Examples of users and queries for ImpleMATE.

Type of user	Requirement	1 ^st^ level – Query for implementation challenge	2 ^nd^ level – Comprehensive implementation support (all data collected fed into Learning Implementation System)
Hospital cardiac department manager	To identify the most effective strategies for implementing remote pulse oximetry.	What are the optimal strategies to implement remote pulse oximetry, and what key barriers hinder patient adoption?	Interacts with ImpleMATE for guided implementation support as part of a continued professional development project.
Hospital CEOs and change management team	To scale and evaluate a precision oncology clinic in routine healthcare.	How can we effectively integrate a precision oncology clinic into existing workflows to improve patient outcomes in hospital settings?	• Change managers receive ImpleMATE support to collect context specific data. • Provide analysis on operational changes, resource allocations, and policy amendments. • Support to develop strategies.
Research team	Understand evidence to introduce whole-body MRI cancer screening in Li-Fraumeni Syndrome.	How effective is whole-body MRI screening, and what behavior changes are needed to support its implementation?	ImpleMATE-guided structured data collection to enable analysis and evaluation.
Department of Health and Aged Care	To run a national campaign to promote HPV self-collection in rural and remote settings.	What contextual and cultural factors influence the successful implementation of HPV self-collection in rural and remote settings?	• Campaign staff to get ImpleMATE training to collect context specific data. • Co-design strategies with ImpleMATE coaching team.

***Establish the acceptability of the interactive web application***

To evaluate and ensure the acceptability of the interactive web application, we will employ ethnographic methods grounded in human-centred design principles. ^82, 83^ These include in-depth structured interviews, contextual inquiry, shadowing, and direct observation of end users in their natural work environments. This approach allows us to capture not only what users say they need, but also what they do in practice, revealing tacit knowledge, workflow constraints, informal processes, and contextual nuances often missed by traditional research methods.

Ethnographic insights will inform iterative co-design sessions where users actively contribute to shaping the application’s interface, features, and functionality. Through participatory design workshops and usability testing cycles, we will evaluate interface intuitiveness, interaction patterns, and overall user satisfaction, applying core user experience (UX) design principles such as simplicity, responsiveness, consistency, and accessibility. ^84– 86^

These methods will help assess users’ experiences and expectations of existing implementation training and coaching methods, identifying gaps and unmet needs. Findings will directly inform platform design to ensure alignment with users’ day-to-day practices, digital literacy levels, organisational cultures, and time/resource constraints.

Additionally, we will develop a structured change management and transition strategy in collaboration with users to support platform adoption. This will include tailored onboarding resources and continuous feedback loops to refine the platform post-deployment. The overall goal is to ensure the platform is not only functional and acceptable but also seamlessly embedded into routine workflows, enabling long-term engagement and trust.

### Activity 4. Design an evaluation framework and roadmap for national integration

Address research questions: SE Q1-2

***An evaluation framework for ImpleMATE***

To evaluate the effectiveness of ImpleMATE and its integration into health systems, we will develop a comprehensive evaluation framework that captures both system-level impact and end-user outcomes. A key component of this evaluation will be the identification of relevant data interfaces such as API that can reflect patient-level outcomes influenced by implementation strategies generated or supported through ImpleMATE. These outcomes may include changes in care pathways, access to evidence-based interventions, and improvements in clinical decision-making resulting from the platform’s decision aid or coaching platform.

We will conduct a mixed-methods pilot evaluation within implementation projects initially focused on cancer genetics and genomics with our multidisciplinary partners, involving implementation scientists, clinicians, data scientists, patients, healthcare managers, and other end users who will use ImpleMATE throughout implementation planning and delivery. The evaluation will compare implementation activities supported by ImpleMATE with conventional implementation approaches that primarily rely on manual evidence extraction, synthesis, expert consultation, and existing implementation planning processes. The pilot evaluation will provide evidence regarding the platform’s technical performance, usability, implementation effectiveness, and organisational readiness, while informing iterative refinement before broader deployment across healthcare settings.

We will collaborate with our multidisciplinary partners to co-develop a set of key performance indicators (KPIs). These KPIs will be designed to assess ImpleMATE’s success from multiple perspectives: ^77^ implementation effectiveness, service delivery, patient outcomes, and system-wide performance.

Primary evaluation outcomes will include the accuracy, relevance, and usefulness of AI-assisted implementation recommendations, usability of the web platform, and implementation planning efficiency. Secondary outcomes will include user satisfaction, trust in AI-assisted recommendations, implementation acceptability, adoption, feasibility, appropriateness, fidelity, organisational readiness, and sustainability, guided by established implementation evaluation frameworks where appropriate. Additional technical performance measures will include AI response time, ontology coverage, and the completeness and consistency of implementation knowledge captured within the platform.

The metrics selected will be guided by the quintuple aim of healthcare improvement: enhancing patient outcomes (e.g., earlier diagnosis, timely treatment, patient-reported satisfaction), improving provider experience (e.g., reduced cognitive burden, support for decision-making), improving health equity, achieving cost-effectiveness, and optimising resource use (e.g., increased speed and quality of evidence-based intervention uptake, reduced duplication of implementation effort, improved allocation of resources).

Quantitative outcomes will be analysed using descriptive statistics together with appropriate comparative analyses where baseline or historical implementation data are available. AI recommendation quality will be assessed through expert ratings of relevance, usefulness, and contextual appropriateness. Qualitative data will be collected through interviews, observations, and user feedback to explore usability, trust, barriers, facilitators, and opportunities for improvement. Quantitative and qualitative findings will be integrated to provide a comprehensive assessment of platform performance and implementation readiness.

***Exploring a national roadmap to integrate ImpleMATE***

The successful implementation of ImpleMATE hinges on its seamless integration into existing healthcare systems in a way that enhances, rather than disrupts, current clinical and organisational workflows. Achieving this requires close collaboration with state-wide health entities and organisations to ensure the platform meets the operational, technical, and regulatory needs of healthcare providers.

To support broader health system integration, ImpleMATE will initially be deployed within secure institutional computing infrastructure and may subsequently be transitioned to secure cloud-based infrastructure where this satisfies institutional security requirements, healthcare privacy standards, and relevant governance requirements. Early in the project, and in alignment with the work of the Principle Setting End User Committee, we will establish a System Integration Advisory Group. This group will bring together technical experts, health service executives, clinicians, IT professionals, and implementation scientists to guide the strategic integration of ImpleMATE across diverse health settings.

Using a process map–guided interview approach, ^87, 88^ we will systematically document and analyse current workflows, information systems, and decision-making structures. This will help identify integration points, organisational readiness, potential barriers, and facilitators. Insights from this process will inform the development of tailored integration strategies that are technically feasible and contextually appropriate.

The roadmap for health system integration will follow a phased implementation approach. The first phase will focus on technical validation of the AI models, ontology, and interactive web platform. The second phase will involve pilot implementation within cancer genetics and genomics services, accompanied by the mixed-methods evaluation described above. Findings from this evaluation will be used to iteratively refine the platform, governance processes, and implementation resources. The final phase will involve planning for broader multi-site implementation and adaptation to additional healthcare domains, informed by predefined technical performance, implementation, usability, and organisational readiness criteria. This staged approach will enable progressive evaluation of ImpleMATE while supporting scalable and sustainable integration into healthcare systems.

### Study status

We have established the ImpleMATE Network, comprising multidisciplinary experts who will form the core of the project’s governance structure. Partnerships have been initiated with collaborators across IT, research technology, cloud infrastructure, and data and AI security to support the development of a secure platform for hosting the web application. Engagements with hospitals and government agencies are also underway to support the co-design process and plan for integration of ImpleMATE into existing healthcare systems. Feasibility assessments and evaluations of locally hosted LLMs for implementation concept identification and extraction have been completed. In parallel, we have explored and shortlisted tools for developing the web application. The first version of the implementation science ontology has also been developed.

## Discussion

ImpleMATE is designed to address the persistent inefficiencies in translating evidence-based healthcare innovations into routine practice by integrating the strengths of implementation science and data science, enhanced through AI. We will build a structured, user-informed platform to accelerate the integration of evidence into healthcare systems at scale. Beginning with the development of a comprehensive ontology to organise implementation knowledge and advancing to the automation of knowledge extraction using LLMs, ImpleMATE has the potential to transform how individuals and organisations identify, access, and apply implementation strategies in real time.

Although this manuscript presents a study protocol, several foundational components of ImpleMATE have already been established, providing confidence in the feasibility of the proposed platform. Preliminary co-design activities involving implementation scientists, data scientists, and information technology experts have informed the overall platform architecture and functional requirements. The platform design has also been informed by our recent work describing how implementation science, data science, and artificial intelligence can be integrated to support multiple facets of implementation. ^89^ These activities have led to the development of an initial implementation ontology framework that captures key implementation science concepts, together with preliminary AI feasibility assessments guided by ontology structure. ^90^ Early web interface prototypes have also been developed to explore user interaction and evidence retrieval workflows. In parallel, stakeholder engagement and co-design activities are underway to inform the responsible AI and governance framework underpinning ImpleMATE, including the identification of user requirements for transparency, human oversight, data governance, and trustworthy AI, ensuring that these principles are embedded into the platform from the outset. While these preliminary activities do not constitute formal evaluation studies and no quantitative performance results are reported in this protocol, they provide proof-of-concept for the proposed methodology and have informed the platform design presented in this manuscript.

The AI-assisted decision aid, underpinned by a robust and expert-informed ontology, is to ensure that decision support is not only AI-generated but also context-aware and aligned with the practical realities of healthcare settings. The platform supports users in navigating unique implementation challenges, making it a powerful resource for implementation practitioners, change managers, healthcare professionals and managers, health innovation and health system researchers, and others engaged in implementation efforts.

ImpleMATE is conceived not as a static solution but as a dynamic learning system. The coaching and data-sharing platform facilitates two-way interactions, enabling users to access tailored implementation support and implementation teams to analyse data through AI-assisted tools to streamline implementation. Our commitment to responsible and ethical AI underpins the ImpleMATE framework. AI transparency, ethical data sharing, and user trust have been prioritised through continuous engagement with a diverse group of stakeholders, including clinicians, consumers, policy-makers, and technical experts.

AI-generated outputs will be grounded in the curated implementation ontology and supporting implementation evidence rather than relying solely on the reasoning capability of the underlying LLM. Where feasible, implementation concepts and recommendations generated by the platform will be accompanied by supporting implementation evidence and ontology concepts to improve transparency and enable users to understand the rationale underpinning AI-generated outputs. Human oversight remains central to the ImpleMATE framework, with implementation scientists and domain experts reviewing AI-generated outputs throughout ontology development, implementation concept extraction, knowledge curation, and platform evaluation before knowledge is incorporated into the implementation knowledge base. As AI-generated recommendations increasingly influence clinical workflows, ImpleMATE is intended to support implementation planning and implementation decision-making rather than provide clinical diagnosis or direct patient management recommendations. We will ensure that decision support is explainable as far as possible, grounded in implementation evidence and expert-informed knowledge, while maintaining transparency about the algorithms and data used in each part of the system. This approach helps maintain trust, ensuring that recommendations are not only grounded in evidence but also aligned with the practical realities of healthcare settings and governed by human oversight.

While ImpleMATE presents significant opportunities to transform healthcare implementation, we acknowledge key risks that must be actively managed to ensure safe, effective, and ethical adoption. First, data security is a core concern, and it is addressed in Activity 1 through secure data storage, encryption protocols, controlled access approvals, and adherence to established standards and best practices, in collaboration with partners to ensure privacy. Second, the accuracy of AI outputs due to complex implementation scenarios and models’ performance is another concern. Activities 2 and 3 involve mitigation strategies including continuous validation, expert feedback, and human review processes to fine-tune models and adjust effective prompts to maintain alignment with evidence-based practices. In addition, AI performance will be monitored across diverse implementation contexts to identify potential systematic bias, with expert review and user feedback informing ongoing refinement of prompts, ontology content, and evidence curation processes to improve robustness, transparency, and generalisability. Third, the risk of building and maintaining user trust and acceptance is supported across all activities by co-design approaches, human-centred design principles, and transparent, expert-informed AI recommendations. Fourth, the challenge of navigating regulatory and legal risks is addressed by actively consulting with relevant authorities and following responsible AI practices that comply with data protection and privacy laws. This will be integrated into all the activities. Finally, ensuring successful implementation and adoption, ImpleMATE prioritises inclusive co-design with stakeholders and consumers, builds a robust integration framework, and establishes clear evaluation metrics to proactively identify and address adoption barriers. As part of this, we will use the co-designed evaluation framework developed in Activity 4 to design a trial that evaluate ImpleMATE within the health system. To inform this evaluation, we conducted a PubMed search on clinical genomics implementation, identifying over 1,500 articles published since 2002, with nearly 90% appearing in the last decade. This trend shows no signs of slowing. As a starting point, we will focus the evaluation on cancer genetics and genomics. This will enable us to assess whether more effective and efficient implementation is achieved in comparison to standard approaches.

To support transparency and reproducibility, we intend to disseminate key research outputs arising from ImpleMATE where appropriate. This will include the implementation ontology, de-identified or synthetic example datasets where permissible, and documentation describing the AI evaluation framework and performance assessment methods. Where ethical, privacy, confidentiality, or intellectual property considerations restrict public release of implementation project data or software components, controlled-access arrangements will be considered in accordance with institutional governance requirements and applicable ethics approvals.

Ultimately, ImpleMATE is designed to form a sustainable learning loop, where real-world data and human input (i.e., human-in-the-loop) continuously inform and improve AI performance. This process ensures that AI inferencing is transparent, grounded in evidence, and aligned with human decision-making. Combining technological innovation with scientific rigor and ethical responsibility, we anticipate that ImpleMATE will offer a transformative, scalable solution to accelerate implementation of evidence-based healthcare innovations to benefit patients.

## Data Availability

No data associated with this article.

## Software availability

No software associated with this article.
