# Supplementary material for: Combining Implementation and Data Sciences to Accelerate Evidence Integration into Healthcare – ImpleMATE
Source: F1000Res. 2026 Sep 10;14:1184. Originally published 2025 Oct 30. [Version 3] doi: 10.12688/f1000research.169999.3 (PMC13589178; doi:10.12688/f1000research.169999.3)
Supplement: Peer review history

# Reviewer report: Reviewer response for version 3

**DOI:** 10.5256/f1000research.209793.r520122

**Published:** 2026-09-18

**Authors:** Tuan D. Pham

**Affiliations:** 1 Queen Mary University of London,, London, UK

The authors have satisfactorily addressed all my concerns.

Is the study design appropriate for the research question?

Yes

Is the rationale for, and objectives of, the study clearly described?

Yes

Are sufficient details of the methods provided to allow replication by others?

Partly

Are the datasets clearly presented in a useable and accessible format?

Not applicable

Reviewer Expertise:

AI in healthcare

I confirm that I have read this submission and believe that I have an appropriate level of expertise to confirm that it is of an acceptable scientific standard.

# Reviewer report: Reviewer response for version 2

**DOI:** 10.5256/f1000research.208120.r440866

**Published:** 2026-08-17

**Authors:** Omid Panahi

**Affiliations:** 1 Centro Escolar University, Manila, Metro Manila, Philippines

The revised manuscript presents a timely and potentially valuable framework for combining implementation science, data science, and AI through the proposed ImpleMATE platform. The rationale and objectives are clearly described, and the overall study design is broadly appropriate for the proposed research questions. The revised manuscript has also substantially strengthened the methodological description, particularly regarding dataset selection, expert annotation, inter-rater agreement, AI evaluation, and the mixed-methods pilot evaluation.

 However, **some methodological issues should still be addressed to make the protocol sufficiently rigorous and reproducible**. The most important remaining concern is that, although the workflow is now described in considerably greater detail, several operational aspects of the AI development and evaluation pipeline remain insufficiently specified for independent replication. The authors should provide, where feasible, more explicit information on the planned model architecture/model selection, key hyperparameters or tuning strategy, prompt-development strategy for LLM components, exact train/validation/test procedures, prevention of data leakage, and the precise performance metrics and decision thresholds that will be used for extraction and recommendation tasks. These details are particularly important because the study's contribution depends substantially on the reliability and reproducibility of the proposed AI pipeline.

 The revised evaluation framework is a clear improvement, with specified outcomes, a mixed-methods pilot design, and quantitative and qualitative analyses.

 Nevertheless, the authors should further clarify the anticipated sample size or recruitment target, participant selection criteria, comparator or baseline condition where applicable, and how missing data and potential sources of bias will be handled. If a formal sample-size calculation is not appropriate for the planned pilot, this should be explicitly justified.

 The manuscript also appropriately strengthens AI safety and human oversight. The evidence-grounded approach, expert review, and explicit distinction between implementation decision support and clinical decision-making are important strengths.

 The authors should nevertheless ensure that these safeguards are translated into measurable evaluation criteria, including how hallucinations, unsupported recommendations, and traceability failures will be detected and reported.

 Finally, the manuscript should provide a clearer reproducibility and sharing plan. The previous review identified the absence of associated data/software as a limitation and recommended sharing the ontology, de-identified or synthetic examples, and evaluation scripts where possible.

 Since this is a protocol and no dataset is currently provided, the dataset-accessibility question is appropriately considered **Not Applicable**; however, a concrete future data/software sharing plan would substantially strengthen transparency and reproducibility.

**Overall, I consider the manuscript substantially improved and potentially suitable for indexing after minor-to-moderate methodological clarification. The key points that must be addressed are the remaining details of the AI evaluation/reproducibility framework, specification of the pilot evaluation and analysis procedures, and a concrete plan for data, ontology, and software/evaluation-script sharing.**

Is the study design appropriate for the research question?

Yes

Is the rationale for, and objectives of, the study clearly described?

Yes

Are sufficient details of the methods provided to allow replication by others?

Partly

Are the datasets clearly presented in a useable and accessible format?

Not applicable

Reviewer Expertise:

Artificial Intelligence in Healthcare; Machine Learning; Digital Dentistry; Clinical AI; Data Science; Digital Health; Evidence-Based Healthcare; Healthcare Innovation; Systematic Reviews and Evidence Synthesis.

I confirm that I have read this submission and believe that I have an appropriate level of expertise to confirm that it is of an acceptable scientific standard, however I have significant reservations, as outlined above.

# Response

**Published:** 2026-09-01

**Authors:** Chi Ho Chan

**Affiliations:** School of Population Health, University of New South Wales Medicine & Health, Sydney, New South Wales, Australia

We appreciate the reviewer’s feedback that the manuscript has substantially improved. We believe these revisions provide a clearer and more reproducible specification of the planned AI pipeline, pilot evaluation, safety assessment, and future sharing arrangements.

**Some methodological issues should still be addressed to make the protocol sufficiently rigorous and reproducible. The most important remaining concern is that, although the workflow is now described in considerably greater detail, several operational aspects of the AI development and evaluation pipeline remain insufficiently specified for independent replication. The authors should provide, where feasible, more explicit information on the planned model architecture/model selection, key hyperparameters or tuning strategy, prompt-development strategy for LLM components, exact train/validation/test procedures, prevention of data leakage, and the precise performance metrics and decision thresholds that will be used for extraction and recommendation tasks. These details are particularly important because the study's contribution depends substantially on the reliability and reproducibility of the proposed AI pipeline.**

**Response:**

 We thank the reviewer for this suggestion. We agree that further operational detail would strengthen the reproducibility of the proposed AI development and evaluation. We now specify the criteria that will inform model selection and provide the key hyperparameters, including learning rate, batch size, training epochs, etc., that will be evaluated during model development (Page 16 – Activity 2, “For the encoder-only concept extractor transformer model…”). The selection will be based on evaluation performance, computational feasibility, suitability for secure local deployment, and potential licensing.

 For the prompt development strategy, we added an iterative and documented process starting with defining task-specific instructions and output schemas to prompt refinement and selection (Pages 17,18 - Activity 2, “Prompt development will follow an iterative, documented process…”)

 We also added more details about train/validation/test procedures and prevention of data leakage (Page 17 – Activity 2, “The training dataset will be used for model development and fine-tuning…”, “The split will be stratified by source type and implementation concept category…”, “…all documents from that project/study will be assigned to the same partition to prevent information leakage…”).

 Regarding decision thresholds, for concept extraction tasks that produce confidence scores, thresholds for accepting extracted concepts will be selected using the validation dataset based on the prespecified performance metrics and subsequently fixed before evaluation on the independent test dataset (Page 17 – Activity 2, “For concept extraction tasks that generate confidence scores…”). For the recommendation tasks, because implementation recommendations are generative outputs rather than conventional classification predictions, a single numerical classification threshold is not directly applicable. Instead, recommendation outputs will be evaluated against traceability, and expert-review criteria (Page 17 – Activity 2, “For generative implementation recommendations… expert assessment of relevance and appropriateness.”).

**The revised evaluation framework is a clear improvement, with specified outcomes, a mixed-methods pilot design, and quantitative and qualitative analyses.**

**Nevertheless, the authors should further clarify the anticipated sample size or recruitment target, participant selection criteria, comparator or baseline condition where applicable, and how missing data and potential sources of bias will be handled. If a formal sample-size calculation is not appropriate for the planned pilot, this should be explicitly justified.**

**Response:**

 We thank the reviewer for this valuable suggestion. We have clarified the pilot evaluation design in Activity 4. We now specify an anticipated recruitment target of approximately 30 end users, purposively sampled to represent diverse user groups. Eligibility is defined as involvement in implementation planning, delivery, evaluation, or evidence translation (Page 22, “The pilot will recruit approximately 30 end users using purposive sampling to represent the intended user groups”).

 We explicitly state that no formal sample size will be undertaken because the pilot is intended to assess feasibility, usability, technical performance, and preliminary signals of effectiveness rather than provide a definitive hypothesis test. The approximately 30-participant target is intended to provide representation across the principal user groups while remaining feasible for qualitative and task-based assessment.

 The comparator has also been clarified. Where feasible, participants will undertake comparable implementation tasks using both conventional approaches and ImpleMATE-supported approaches. Where a within-project comparison is not feasible, baseline or historical implementation data will be used as the comparator (Page 22, “Where feasible, participants will complete comparable tasks using both approaches…”).

 Missing-data procedures and potential biases are now described. Missingness will be summarised by outcome and participant group; analyses will use available data. AI performance outcomes will not be imputed when reference annotations or expert ratings are unavailable. We also identify and describe mitigation of different biases (Pages 22,23, “Missing data and sources of bias will be documented…”). 

**The manuscript also appropriately strengthens AI safety and human oversight. The evidence-grounded approach, expert review, and explicit distinction between implementation decision support and clinical decision-making are important strengths.**

**The authors should nevertheless ensure that these safeguards are translated into measurable evaluation criteria, including how hallucinations, unsupported recommendations, and traceability failures will be detected and reported.**

**Response:**

 Thank you very much for this suggestion. We agree with the reviewer and have added specific procedures for these safeguards in Activity 2. Hallucination and unsupported recommendations are now defined as a factual statement or implementation recommendation that cannot be supported by or traced to the source material and curated ontology. Traceability completeness will be calculated as the proportion of factual claims or recommendations for which the system provides a valid link to the supporting evidence and/or relevant ontology concept. 

 A stratified sample of AI-generated outputs will be independently reviewed by two implementation science experts, with disagreements resolved through consensus or senior adjudication. We will report the number and proportion of hallucinations, unsupported recommendations, and traceability failures, with 95% confidence intervals where sample size permits. For the pilot decision-support evaluation, outputs must meet the evidence-support and traceability requirements and the prespecified expert-rating criteria to be considered acceptable; outputs failing these criteria will be flagged for review and excluded from the knowledge base until corrected and revalidated. (Page 18, “AI safety outputs will be assessed and reported using prespecified measurable indicators…”)

**Finally, the manuscript should provide a clearer reproducibility and sharing plan. The previous review identified the absence of associated data/software as a limitation and recommended sharing the ontology, de-identified or synthetic examples, and evaluation scripts where possible.**

**Since this is a protocol and no dataset is currently provided, the dataset-accessibility question is appropriately considered Not Applicable; however, a concrete future data/software sharing plan would substantially strengthen transparency and reproducibility.**

**Response:**

 We appreciate this suggestion to strengthen transparency and reproducibility. We have strengthened the reproducibility and sharing plan in the Discussion section. The planned outputs now include versioned releases of the implementation ontology and its documentation, annotation guidelines, data dictionaries, prompt templates, de-identified or synthetic example datasets where permissible, evaluation scripts, analysis code, and selected software components. Model specifications and configuration information will be documented to support replication using compatible locally hosted models. (Pages 25,26 “To support transparency and reproducibility…”)

# Reviewer report: Reviewer response for version 1

**DOI:** 10.5256/f1000research.187409.r428532

**Published:** 2025-12-30

**Authors:** Tuan D. Pham

**Affiliations:** 1 Queen Mary University of London,, London, UK

The manuscript presents ImpleMATE, an AI-powered implementation science platform aimed at accelerating the translation of research evidence into healthcare practice.  The concept integrates implementation science, data science, and artificial intelligence (AI) within a Learning Health System (LHS) framework to establish continuous feedback and improvement cycles.  The project will employ co-design and co-production approaches, involving stakeholders in healthcare and data science to develop a structured ontology, an AI-driven knowledge extraction system, and an interactive web platform to support implementation decision-making.  The proposed system aspires to address the long-standing issue of delayed evidence integration into clinical practice, particularly focusing initially on cancer genetics and genomics.

**Strengths**

1. Innovative Integration: The paper successfully combines implementation science and data science into a coherent conceptual framework, bridging theoretical and applied domains.
2. Comprehensive Structure: The manuscript is systematically organized around the LHS cycle, enhancing readability and logical flow.
3. Methodological Transparency: Detailed methodological descriptions are provided for data processing, ontology development, AI system design, and evaluation planning.
4. Ethical Considerations: The inclusion of dedicated ethical governance (e.g., End User Committee and ImpleMATE Ethics Principles) demonstrates foresight and responsible innovation.
5. Stakeholder Involvement: The co-design and co-production approach aligns well with human-centred design principles and strengthens translational potential.

**Major Issues**

1. Empirical Validation Plan:  The protocol outlines AI development and ontology creation but lacks sufficient detail on how empirical validation will be quantitatively measured beyond standard metrics (precision, recall, F1-score).  The authors may include a validation study design detailing sample sizes, validation datasets, and expected benchmarks compared with existing manual or semi-automated approaches. Clarify how “success” in the AI’s performance will be defined statistically and pragmatically.
2. Evaluation Framework Specificity: The evaluation framework for national integration is conceptually described but remains too generic.  Define specific, measurable KPIs (e.g., reduction in implementation time, accuracy of decision support recommendations, improvements in patient outcomes). Include a timeline or phase plan (e.g., pilot, scale-up, evaluation).
3. Data Governance and Security Implementation:  While ethical principles are well discussed, there is limited technical detail on how privacy, consent, and data security will be enforced, particularly for sensitive genomic data.  Add a technical appendix or methods subsection specifying: Data encryption standards, federated data storage options, access control and logging mechanisms, governance under relevant data protection acts (e.g., GDPR, Australian Privacy Principles).
4. Ontology Development and Interoperability:  The proposed ontology (Figure 3, page 9) is conceptual but requires clearer definition of ontology scope and interoperability mechanisms with existing biomedical ontologies (e.g., SNOMED CT, OBO Foundry ontologies).  The authors should describe the mapping process to established terminologies, including ontology evaluation criteria such as completeness, consistency, and usability across multiple healthcare domains.
5. AI Transparency and Bias Mitigation:  The manuscript notes the risk of bias and hallucination in LLMs but provides limited description of bias monitoring mechanisms.  Specify procedures for bias auditing, model retraining protocols, and inclusion of diverse datasets to reduce cultural or contextual bias in implementation recommendations.
6. Lack of Preliminary Pilot Data:  Although it is a protocol, references to completed “feasibility assessments” and “first version of ontology” suggest early work has been done. However, no preliminary outcomes or examples are provided.  Please summarize these preliminary findings to support feasibility and establish proof-of-concept credibility.

**Minor Comments**

1. Figures and Visual Clarity:  Figures 1–4 are informative but could benefit from clearer legends and scalable resolution for digital publication.
2. Terminology Consistency:  Ensure consistent use of abbreviations (e.g., “LLMs,” “AI-powered implementation science platform”) across sections.
3. Formatting:  The Methods section is lengthy; consider condensing repetitive explanations of co-design principles to improve readability.
4. Discussion Section:  Add a brief comparison to existing AI-enabled implementation platforms (if any) to highlight novelty and justify distinctiveness of ImpleMATE.

Is the study design appropriate for the research question?

Yes

Is the rationale for, and objectives of, the study clearly described?

Yes

Are sufficient details of the methods provided to allow replication by others?

Partly

Are the datasets clearly presented in a useable and accessible format?

Not applicable

Reviewer Expertise:

AI and data science in medicine and life science.

I confirm that I have read this submission and believe that I have an appropriate level of expertise to confirm that it is of an acceptable scientific standard, however I have significant reservations, as outlined above.

# Response

**Published:** 2026-08-06

**Authors:** Chi Ho Chan

**Affiliations:** School of Population Health, University of New South Wales Medicine & Health, Sydney, New South Wales, Australia

We sincerely thank the reviewer for the careful evaluation of our manuscript and for the constructive and insightful comments. We appreciate the time and effort invested in reviewing our work. We have carefully addressed all comments and have revised the manuscript accordingly.

**Major Issues**

 1. Empirical Validation Plan: The protocol outlines AI development and ontology creation but lacks sufficient detail on how empirical validation will be quantitatively measured beyond standard metrics (precision, recall, F1-score).  The authors may include a validation study design detailing sample sizes, validation datasets, and expected benchmarks compared with existing manual or semi-automated approaches. Clarify how “success” in the AI’s performance will be defined statistically and pragmatically.

**Response:**

 We thank the reviewer for this valuable suggestion. We agree that the empirical validation plan should be more explicit. We have expanded **Activity 2 **to describe the validation study design, including the annotated dataset, train/validation/test split, comparison against manual expert annotation, statistical evaluation metrics, and predefined performance targets. We also clarify that AI performance will be assessed using both quantitative accuracy metrics and pragmatic measures of usefulness for implementation researchers. ( **Pages 16 and 17**, Section – AI performance evaluation metrics, quality assessment and monitoring, “The annotated dataset will be…”, “Performance evaluation will also include…”, “Pragmatic success criteria will include…”)

 2. Evaluation Framework Specificity: The evaluation framework for national integration is conceptually described but remains too generic.  Define specific, measurable KPIs (e.g., reduction in implementation time, accuracy of decision support recommendations, improvements in patient outcomes). Include a timeline or phase plan (e.g., pilot, scale-up, evaluation).

**Response:**

 We appreciate the reviewer's comment. We have substantially strengthened **Activity 4 (Design an evaluation framework and roadmap for national integration)** by providing a more detailed evaluation framework. We now describe a mixed-methods pilot evaluation within implementation projects initially focused on cancer genetics and genomics, including the target participants, comparison with conventional implementation approaches, and an iterative evaluation process prior to broader deployment ( **Page 21**, “We will conduct a mixed-methods pilot evaluation…”). We also define primary and secondary evaluation outcomes for the KPIs, including AI recommendation quality, implementation planning efficiency, usability, user satisfaction, implementation outcomes, and organisational readiness ( **Page 21**, “Primary evaluation outcomes will include…”). We have also expanded the evaluation framework to include both quantitative and qualitative analysis approaches ( **Page 21**, “Quantitative outcomes will be analysed using…”), together with a phased roadmap from pilot evaluation to broader health system integration ( **Page 22**, “The roadmap for health system integration will follow a phased implementation approach…”).

 3. Data Governance and Security Implementation:  While ethical principles are well discussed, there is limited technical detail on how privacy, consent, and data security will be enforced, particularly for sensitive genomic data.  Add a technical appendix or methods subsection specifying: Data encryption standards, federated data storage options, access control and logging mechanisms, governance under relevant data protection acts (e.g., GDPR, Australian Privacy Principles).

**Response:**

 We thank the reviewer for this valuable suggestion. We agree that the original manuscript focused primarily on the ethical principles underpinning ImpleMATE and did not sufficiently describe the operational implementation of data governance and security. Therefore, we have expanded **Activity 1 (Ethical sharing of project data and responsible use of AI)** to provide additional technical detail on the management of sensitive implementation project and health data.

 The revised manuscript now describes the secure data management framework underpinning ImpleMATE, including data de-identification prior to AI processing, secure institutional computing infrastructure, the use of locally hosted AI models during development and evaluation, encryption of data during storage and transmission, role-based access control, audit logging, and data retention procedures ( **Page 14**, “To operationalise these governance principles…”). We also clarify that governance will comply with institutional ethics approvals, the State and Federal Australian Privacy Laws. Furthermore, we describe that future deployment to cloud-based environments will only occur where the infrastructure satisfies institutional security, privacy, and governance requirements ( **Page 11**, “The platform will initially operate within…”) ( **Page 15**, “The use of locally hosted models within secure institutional computing infrastructure…”) ( **Page 22**, “To support broader health system integration, ImpleMATE will initially be deployed within secure institutional computing infrastructure…”).

 4. Ontology Development and Interoperability:  The proposed ontology (Figure 3, page 9) is conceptual but requires clearer definition of ontology scope and interoperability mechanisms with existing biomedical ontologies (e.g., SNOMED CT, OBO Foundry ontologies).  The authors should describe the mapping process to established terminologies, including ontology evaluation criteria such as completeness, consistency, and usability across multiple healthcare domains.

**Response:**

 We thank the reviewer for this constructive suggestion. We agree that the original manuscript did not sufficiently describe the scope and interoperability of the proposed implementation ontology. We have expanded the **Activity 1 (An implementation schema to guide the development of ontology)**.

 We now clarify that the ImpleMATE ontology will initially focus on implementation science concepts relevant to cancer genetics and genomics while being designed as a modular and extensible framework that can be progressively expanded to other healthcare domains ( **Page 12**, “This schema will establish the scope of the ImpleMATE ontology by defining…”). We have also strengthened the description of interoperability by explaining that, where appropriate, implementation concepts will be aligned with or reference established biomedical terminologies and ontologies to facilitate interoperability with existing health information systems, and avoid duplication of established biomedical knowledge ( **Page 12**, “Where appropriate, implementation concepts will align with…”).

 In addition, we have expanded the manuscript to describe the iterative evaluation of the ontology using both technical and expert-informed criteria. Technical evaluation will assess logical consistency, completeness, interoperability, scalability, and extensibility, while expert evaluation involving implementation scientists, clinicians, healthcare managers, and end users will assess the ontology's accuracy, relevance, usability, and practical value for supporting implementation research and practice ( **Page 13**, “The ontology will be refined iteratively throughout the project using both technical and expert-informed evaluation…”).

 5. AI Transparency and Bias Mitigation:  The manuscript notes the risk of bias and hallucination in LLMs but provides limited description of bias monitoring mechanisms.  Specify procedures for bias auditing, model retraining protocols, and inclusion of diverse datasets to reduce cultural or contextual bias in implementation recommendations.

**Response:**

 We thank the reviewer for this suggestion. We agree that the original manuscript would benefit from a clearer description of how AI transparency and bias mitigation will be operationalised within ImpleMATE. We have expanded **Activity 2 (Develop and validate an AI system to automate ontology creation and deliver real-time solutions to implementation challenges)** to strengthen the description of these processes.

 We clarify that ImpleMATE adopts an evidence-grounded approach, where implementation concept extraction and AI-generated outputs are informed by the curated implementation ontology and supporting implementation evidence, rather than relying solely on the reasoning capability of the underlying large language model. Where feasible, AI-generated outputs will be linked to the corresponding ontology concepts and supporting evidence used during inference, improving transparency and enabling users to understand the rationale underpinning AI-assisted outputs ( **Pages 14-17**).

 We have also strengthened the description of human-in-the-loop validation, clarifying that implementation scientists and domain experts will review AI-generated outputs throughout ontology development, implementation concept extraction, knowledge curation, and platform evaluation before knowledge is incorporated into the implementation knowledge base ( **Page 16**, “Importantly, ontology updates and knowledge curation will remain subject to expert review through a human-in-the-loop process…”) ( **Page 17**, “Human-in-the-loop review will be incorporated throughout ontology development…”).

 To address bias mitigation, the revised manuscript now specifies that AI performance will be monitored across diverse healthcare settings, intervention types, implementation contexts, and populations to identify potential systematic bias. Expert review, user feedback, and ongoing performance evaluation will inform refinement of prompts, ontology content, and evidence curation processes to improve robustness, transparency, and generalisability ( **Page 16**, “Implementation scientists will review AI-generated outputs throughout model development to identify incorrect, incomplete, or potentially biased implementation concepts…”) ( **Page 17**, “Performance monitoring will also assess the consistency of concept extraction across diverse healthcare settings…”). We also explicitly clarify that ImpleMATE is designed to support implementation decision-making, rather than provide clinical diagnosis or direct patient management recommendations, with final decisions remaining the responsibility of implementation teams and healthcare professionals ( **Page 17**, “ImpleMATE is intended to support implementation decision-making…”).

 6. Lack of Preliminary Pilot Data:  Although it is a protocol, references to completed “feasibility assessments” and “first version of ontology” suggest early work has been done. However, no preliminary outcomes or examples are provided.  Please summarize these preliminary findings to support feasibility and establish proof-of-concept credibility.

**Response:**

 We thank the reviewer for this suggestion. We have expanded the **Discussion** to include a summary of the preliminary development work that underpins ImpleMATE.

 We now describe the completion of initial co-design activities involving implementation scientists, data scientists, and information technology experts, which informed the platform architecture and functional requirements. We also describe the development of an initial implementation ontology framework, preliminary AI feasibility assessments guided by ontology structure, prompt engineering strategies, implementation concept extraction workflows, and the development of early web interface prototypes for evidence retrieval and implementation support.

 We further clarify that these preliminary activities were undertaken to establish the technical and methodological feasibility of the proposed platform and to inform refinement of the platform design, rather than to formally evaluate system performance. Therefore, no quantitative performance results are presented in this protocol. We believe these additions provide appropriate proof-of-concept evidence while maintaining the manuscript's focus as a study protocol ( **Page 23**, “Although this manuscript presents a study protocol…”).

**Minor Comments**

 1. Figures and Visual Clarity:  Figures 1–4 are informative but could benefit from clearer legends and scalable resolution for digital publication.

**Response:**

 Thank you for this suggestion. We have revised the figure legends for better clarity and will provide higher-resolution figures suitable for publication.

 2. Terminology Consistency:  Ensure consistent use of abbreviations (e.g., “LLMs,” “AI-powered implementation science platform”) across sections.

**Response:**

 We thank the reviewer for identifying this issue. We have reviewed the manuscript and standardised terminology throughout, including consistent use of abbreviations such as AI, LLMs, and ImpleMATE as an AI-assisted implementation science platform.

 3. Formatting:  The Methods section is lengthy; consider condensing repetitive explanations of co-design principles to improve readability.

**Response:**

 Thank you for this suggestion. We have carefully reviewed the Methods section to reduce unnecessary repetition and improve readability where appropriate.

 4. Discussion Section:  Add a brief comparison to existing AI-enabled implementation platforms (if any) to highlight novelty and justify distinctiveness of ImpleMATE.

**Response:**

 We appreciate this recommendation. We carefully considered adding a comparison with existing AI-enabled implementation platforms. To the best of our knowledge, there are currently no published AI-enabled implementation science platforms that provide functionality comparable to ImpleMATE by integrating implementation science, AI-assisted implementation decision support, implementation coaching, ontology-based knowledge management, and continuous learning within a Learning Health System. As we were unable to identify a directly comparable platform, we did not include a comparison in the revised manuscript to avoid potentially misleading comparisons.

# Reviewer report: Reviewer response for version 1

**DOI:** 10.5256/f1000research.187409.r440859

**Published:** 2025-12-16

**Authors:** Yogeesh Nijalingappa

**Affiliations:** 1 Government First Grade College, Tumkur, India

**Summary**

 This manuscript presents a **protocol** for developing **ImpleMATE**, an AI-supported implementation science platform that uses an ontology + LLM-based extraction to support implementation planning and learning health system cycles. The topic is important and timely, and the overall concept is clear.

 Responses to the core questions

- **Rationale and objectives:** **Yes** — the problem statement and aims are clear.
- **Study design appropriate:** **Partly** — the plan is reasonable for a protocol, but the evaluation design is not yet described in a testable way.
- **Methods replicable:** **Partly** — the workflow is described at a high level, but key operational details are missing.
- **Datasets accessible:** **Not applicable (protocol)** — no dataset/software is provided.

Major comments (must be addressed to be scientifically sound)

1. **Evaluation plan is not sufficiently defined**
  The manuscript mentions KPIs and broad goals, but it does not specify a clear evaluation study design.
**Must add:** primary outcomes, sample/setting, comparison condition (if any), study type (pilot/pre-post/mixed methods), and a short analysis plan.
2. **Insufficient methodological detail for replication (AI + annotation)**
  The protocol states that expert annotation will be used, but does not describe document inclusion criteria, annotation guidelines, inter-rater reliability targets, adjudication, and train/test splits.
**Must add:** clear dataset selection rules, annotation process, reliability metrics, and evaluation metrics for extraction/recommendation performance.
3. **LLM safety/traceability needs stronger operational detail**
  Hallucination is acknowledged, but there is no detailed mitigation approach.
**Must add:** evidence-grounded outputs (citations to source passages/ontology nodes), human-in-the-loop review points, and explicit limitations that the system supports implementation planning (not clinical decision-making).
4. **Data governance and ethics need more concrete procedures**
  Governance principles are described, but practical details for using real-world project data are limited.
**Must add:** consent/ethics pathway, de-identification workflow, access control, audit logging, and data retention policies.
5. **Reproducibility and sharing plan is missing**
  The paper states no associated data/software. For an AI platform protocol, this weakens reproducibility.
**Must add:** at least an intention to share the ontology, sample de-identified/synthetic examples, and evaluation scripts (or provide controlled-access details).

**Minor comments**

- Please check an affiliation line that appears incorrect (“Zurich, Swaziland”).
- Improve consistency in terminology across sections (platform vs decision aid vs coaching tool).

**Recommendation**

**Major revisions.** The idea is strong, but the manuscript requires clearer evaluation design, replicable methods, and stronger AI/data governance detail to be scientifically rigorous.

Is the study design appropriate for the research question?

Partly

Is the rationale for, and objectives of, the study clearly described?

Yes

Are sufficient details of the methods provided to allow replication by others?

Partly

Are the datasets clearly presented in a useable and accessible format?

Not applicable

Reviewer Expertise:

Related

I confirm that I have read this submission and believe that I have an appropriate level of expertise to confirm that it is of an acceptable scientific standard, however I have significant reservations, as outlined above.

# Response

**Published:** 2026-08-06

**Authors:** Chi Ho Chan

**Affiliations:** School of Population Health, University of New South Wales Medicine & Health, Sydney, New South Wales, Australia

We sincerely thank the reviewer for the careful evaluation of our manuscript and for the constructive and insightful comments. We appreciate the time and effort invested in reviewing our work. We have carefully addressed all comments and have revised the manuscript accordingly.

**Major comments**

**1. Evaluation plan is not sufficiently defined**

 The manuscript mentions KPIs and broad goals, but it does not specify a clear evaluation study design.

**Must add:** primary outcomes, sample/setting, comparison condition (if any), study type (pilot/pre-post/mixed methods), and a short analysis plan.

**Response:**

 We thank the reviewer for this helpful suggestion. We agree that the original manuscript did not provide sufficient evaluation plan. We have now substantially expanded **Activity 4 (Design an evaluation framework and roadmap for national integration)** to specify the evaluation methodology, including the primary and secondary evaluation outcomes for the KPIs, including AI recommendation quality, implementation planning efficiency, usability, user satisfaction, implementation outcomes, and organisational readiness  ( **Page 21**, “Primary evaluation outcomes will include the accuracy, relevance…”), setting and study design ( **Page 21**, “We will conduct a mixed-methods pilot evaluation within implementation projects initially focused on cancer genetics and genomics…”), and quantitative and qualitative analysis plan ( **Page 21**, “Quantitative outcomes will be analysed using…”). We also describe how the findings will inform iterative refinement prior to broader health system implementation ( **Page 22**, “The roadmap for health system integration will follow a phased implementation approach… Findings from this evaluation will be used to iteratively refine the platform…”).

**2. Insufficient methodological detail for replication (AI + annotation)**

 The protocol states that expert annotation will be used, but does not describe document inclusion criteria, annotation guidelines, inter-rater reliability targets, adjudication, and train/test splits.

**Must add:** clear dataset selection rules, annotation process, reliability metrics, and evaluation metrics for extraction/recommendation performance.

**Response:**

 We thank the reviewer for this helpful suggestion. We agree that additional methodological detail will improve the reproducibility of the proposed AI development and evaluation process. We have expanded **Activity 2** to provide a more detailed description of dataset selection ( **Page 14**, “Documents will be purposively selected to represent a broad range of implementation studies…”), expert annotation ( **Page 14**, “Annotation will be undertaken using a standardised annotation guideline…”), and model evaluation ( **Pages 16 and 17**, “The annotated dataset will be randomly divided into training…performance metrics, including precision…”).

 We now describe the document selection criteria used to construct the gold-standard dataset, including the inclusion of diverse implementation studies, healthcare settings, intervention types, and implementation contexts. We have also clarified that annotation will be conducted using a standardised annotation guideline derived from the implementation ontology, with pilot annotation undertaken to refine the guideline before full annotation.

 To strengthen annotation quality, we specify that each document will be independently annotated by at least two implementation science experts, with inter-rater agreement assessed using Cohen's kappa and disagreements resolved through consensus or adjudication by a senior implementation scientist. We further clarify that the final consensus annotations will form the gold-standard dataset used for AI development and evaluation ( **Page 15**, “Each document will be independently annotated by at least two implementation science experts…”).

**3. LLM safety/traceability needs stronger operational detail**

 Hallucination is acknowledged, but there is no detailed mitigation approach.

**Must add:** evidence-grounded outputs (citations to source passages/ontology nodes), human-in-the-loop review points, and explicit limitations that the system supports implementation planning (not clinical decision-making).

**Response:**

 We thank the reviewer for this valuable suggestion. We have strengthened the manuscript to provide additional operational detail regarding LLM safety and traceability. We now clarify that AI-generated outputs will be grounded in the curated implementation ontology, with implementation recommendations linked to the supporting implementation evidence and relevant implementation concepts wherever appropriate ( **Page 16**, “To promote trustworthy AI, ImpleMATE will adopt an evidence-grounded approach…”). We have also expanded the description of the human-in-the-loop review process, clarifying that expert validation will occur throughout ontology development, implementation concept extraction, knowledge curation, and platform evaluation before AI-generated outputs are incorporated into the knowledge base or presented through the web application ( **Page 16**, “ontology updates and knowledge curation will remain subject to expert review through a human-in-the-loop process…”; **Page 17**, “Human-in-the-loop review will be incorporated throughout ontology development…”). Finally, we explicitly state that ImpleMATE is intended to support implementation planning and implementation decision support rather than clinical decision-making, and that all AI-generated outputs require appropriate human oversight ( **Page 17**, “Importantly, ImpleMATE is intended to support implementation decision-making rather than provide clinical diagnosis or direct patient management recommendations…”).

**4. Data governance and ethics need more concrete procedures**

 Governance principles are described, but practical details for using real-world project data are limited.

**Must add:** consent/ethics pathway, de-identification workflow, access control, audit logging, and data retention policies.

**Response:**

 We appreciate this suggestion. We have strengthened the description of data governance procedures in **Activity 1 (Ethical sharing of project data and responsible use of AI)** by providing additional detail regarding the use of real-world implementation project data. We now clarify that implementation project data will only be used following the relevant institutional ethics and governance approvals and participant consent where required. We also describe the de-identification workflow, role-based access controls, audit logging, and institutional data retention and disposal procedures ( **Page 14**, “ImpleMATE will implement a secure data management framework that protects sensitive implementation project and health data…”). These additions complement the existing description of secure computing infrastructure and privacy safeguards ( **Page 14**, “ImpleMATE will prioritise locally hosted AI models operating within secure computing environments…”).

**5. Reproducibility and sharing plan is missing**

 The paper states no associated data/software. For an AI platform protocol, this weakens reproducibility.

**Must add:** at least an intention to share the ontology, sample de-identified/synthetic examples, and evaluation scripts (or provide controlled-access details).

**Response:**

 We thank the reviewer for this recommendation. We agree that transparency and reproducibility are important considerations for AI-enabled research. Therefore, we have added a statement to the **Discussion** outlining our intention to disseminate key research outputs arising from ImpleMATE, including the implementation ontology, de-identified or synthetic example datasets where appropriate, and documentation describing the AI evaluation framework and performance metrics. We also clarify that, where implementation project data or software components cannot be publicly released because of ethical, privacy, or intellectual property considerations, access will be managed through appropriate controlled-access arrangements in accordance with institutional governance requirements and ethics approvals ( **Page 24**, “To support transparency and reproducibility…”).

**Minor comments**

 • Please check an affiliation line that appears incorrect (“Zurich, Swaziland”).

**Response:**

 Thank you for identifying this error. The affiliation has been corrected to "Zurich, Switzerland."

 • Improve consistency in terminology across sections (platform vs decision aid vs coaching tool).

**Response:**

 We appreciate this observation. We have reviewed the manuscript and standardised terminology throughout to consistently describe ImpleMATE as an AI-assisted implementation science platform comprising an AI-assisted decision aid and implementation coaching and data sharing platform.
